# Artificial intelligence applied to post-resuscitation ECGs for early prognostication after out-of-hospital cardiac arrest

**DOI:** 10.3389/fcvm.2026.1765751

**Published:** 2026-05-11

**Authors:** Francesca R. Gentile, Syed Taimoor Hussain Shah, Michela Sperti, Konstantinos Panagiotopoulos, Roberto Primi, Leila Ulmanova, Alessia Currao, Sara Bendotti, Enrico Baldi, David N. Bauer, Silvia M. Pontremoli, Gianluca Marconi, Marco A. Deriu, Simone Savastano

**Affiliations:** 1 Cardiac Arrest and Resuscitation Research Team (RESTART), Fondazione IRCCS Policlinico San Matteo, Pavia, Italy; 2 Yale New Haven Health System, Bridgeport Hospital, Bridgeport, CT, United States; 3 PolitoBIOMed Lab, Department of Mechanical and Aerospace Engineering, Politecnico di Torino, Torino, Italy; 4 Division of Cardiology, Fondazione IRCCS Policlinico San Matteo, Pavia, Italy; 5 Department of Public Health, Experimental and Forensic Medicine, University of Pavia, Pavia, Italy; 6 Department of Internal Medicine and Medical Therapy, University of Pavia, Pavia, Italy; 7 Agenzia Regionale Emergenza Urgenza della Regione Lombardia (AREU), Milano, Italy

**Keywords:** 12-lead ECG, artificial intelligence (AI), deep neural network (DNN), machine learning, neurological outcome, out-of-hospital cardiac arrest (OHCA), pre-hospital care, prognostication

## Abstract

**Background:**

There are limited tools for early outcome prediction after Out-of-Hospital Cardiac Arrest (OHCA). This study aimed to evaluate whether a machine learning model could help to predict neurological outcome using the 12-lead ECG acquired on scene following return of spontaneous circulation (ROSC).

**Methods:**

We conducted a retrospective analysis of prospectively collected post-ROSC ECGs from the Lombardy Cardiac Arrest Registry (January 2015-December 2023). The study included all the patients resuscitated from OHCA with a 12-lead ECG acquired on scene after ROSC. A deep neural network model was developed, validated, and tested using features extracted from these ECGs via computer vision techniques, incorporating patient age, sex, initial rhythm, and ROSC-ECG time. The dataset was split into training (80%), validation (10%), and independent testing (10%) sets. Model performance was evaluated using accuracy, sensitivity, specificity, positive predictive value (PPV), negative predictive value (NPV), AUROC (Area Under the Receiver Operating Characteristic curve), Matthews Correlation Coefficient (MCC), and SHAP (Shapley Additive exPlanations) values for interpretability.

**Results:**

We included 976 post-ROSC ECGs (641 poor and 335 favorable neurological outcomes). The model achieved an accuracy of 80.8%, sensitivity of 86.1%, specificity of 74.3%, PPV of 80.4%, and NPV of 81.2%, with an MCC of 0.61 and an AUROC of 0.86.

**Conclusions:**

To our knowledge, this is the first machine learning model utilizing post-ROSC 12-lead ECG data to evaluate its association with neurological outcome immediately after ROSC. Its application in the pre-hospital setting may provide additional information to support clinical decision-making regarding transport strategies and post-resuscitation care planning following OHCA.

## 1 Introduction

Despite progress in cardiopulmonary resuscitation (CPR) and post-resuscitation care, survival at discharge of patients resuscitated from an out-of-hospital cardiac arrest (OHCA) remains extremely poor and generally occurs in less than 10% of patients (1). While mortality from circulatory failure prevails in the first days after return of spontaneous circulation (ROSC), the most common cause of in-hospital death is severe post-ROSC brain injury resulting in withdrawal of life-sustaining treatment based on unfavorable neurological prognosis (2). The prediction in case of bad neurological outcome is currently based on a complex multiparametric prognostication approach (3) which uses clinical examination, biomarkers and imaging techniques. In the past, different prognostic scores have been developed in order to identify patients at high risk of death or poor neurological outcome (4–7). However, they are all based on variables available only after hospital admission, so they prevent prognostication from beginning in the pre-hospital setting. This is not a trivial detail because emergency medical system (EMS) personnel, besides performing advanced resuscitation, also have to triage and transport the patient to the most appropriate center and communicate with families about the patient's condition. Such a delicate and time-sensitive process requires quick and effective decision making and has inspired different pre-hospital scores (8–10). Notably, none of them rely on factors that reflect patients' individual conditions, rather they are based on circumstantial factors related to the OHCA event itself.

After a cardiac arrest a 12-lead electrocardiogram (ECG) is acquired in the pre-hospital setting, as recommended by guidelines, with the aim to disclose acute coronary syndrome (ACS) with ST-segment elevation which represents the main indication for urgent coronary angiography (11–13). The prognostic impact of the ECG has been demonstrated in several cardiac and non-cardiac conditions (14–17). Few studies have investigated the relationship between ECG and outcomes in OHCA survivors (18, 19), and only one recent study showed that post-ROSC ECG has a prognostic role in predicting early mortality in OHCA patients (20). However, that study was based on the identification of traditional ECG features, readable by clinicians. The application of artificial intelligence (AI) to the ECG has increased dramatically (21) showing fascinating data related to the prediction of myocardial infarction, atrial fibrillation, valvular diseases and sudden cardiac death (22). The only existing application of AI specifically to post-ROSC ECG is based on the prediction of the culprit lesion after OHCA (23).

The aim of this study is to apply artificial intelligence to post-ROSC 12-lead ECGs to assess their association with neurological outcomes following out-of-hospital cardiac arrest.

## 2 Materials and methods

### 2.1 Study type

This is a multicenter, retrospective analysis of post-ROSC ECGs prospectively collected in the longitudinal Utstein-based OHCA registry “*Lombardia CARe”* (ClinicalTrials.gov ID: NCT03197142). The *Lombardia CARe* registry collects the operational and clinical data for all OHCA patients occurring in 7 Italian provinces in northern Italy. A complete description of the characteristics collected in the registry is provided in the Supplementary Table S1.

### 2.2 Data collection, study population and ECG

In this study, all the patients resuscitated from an OHCA occurred between January 1st, 2015 and December 31st, 2023, regardless of the etiology, were considered eligible for inclusion.

### 2.3 Inclusion and exclusion criteria

We included all the patients with known sex, age who sustained ROSC, had documented survival status and neurological outcome at hospital discharge (expressed by Cerebral Performance Category scale - CPC), and for whom the first post-ROSC 12-lead ECG was available in a digital format (e.g.,pdf, JPEG). Patients were excluded if they did not meet the eligibility criteria or if their post-ROSC ECG was unreadable due to artifacts or was suboptimal for machine learning (ML) analysis (Supplementary Figure S1). The detailed reasons for ECG exclusion with the sample breakdown are shown in Supplementary Table S2. Since ECGs were available as image-based documents rather than raw waveform files, we extracted computer-vision descriptors (SIFT and bag-of-visual-words) directly from the images; therefore, acquisition sampling frequency was not used. To standardize input quality, PDF ECGs were converted to images at 300 DPI prior to feature extraction.

### 2.4 Electrocardiogram acquisition

Among all the 12-lead ECGs acquired in the field after ROSC and subsequently transmitted for evaluation by the regional Emergency Operations Center (Sala Operativa Regionale dell'Emergenza Urgenza - SOREU), the first ECG available for every patient is stored pseudo-anonymously in the *Lombardia CARe* registry in PDF format. Each ECG, collected during the study period from the provinces of Pavia, Lodi, Cremona, Mantova, Brescia, Varese, and Como, was transferred to the Polytechnic University of Turin for the development of a machine-learning model.

### 2.5 Outcome definitions

The neurological outcome was graded at discharge using the Cerebral Performance Category (CPC) scale: CPC 1 represents normal neurological function, CPC 2 moderate impairment but with independent living, CPC 3 severe impairment, CPC 4 persistent coma and CPC 5 cerebral death. The CPC scale is often dichotomized into good neurological outcome (CPC ≤2) and poor neurological outcome (CPC>2). For this study, we used a binary classification based on CPC score and survival status at discharge: Class 0 for individuals who survived with good neurological outcome (CPC≤2) and Class 1 for patients with death or poor neurological outcome (CPC>2) at hospital discharge.

### 2.6 Visual feature extraction and model validation

After the preprocessing phase (supplementary Section “*ECG files preprocessing*”), visual features were extracted from the post-ROSC ECGs with the help of two well-known computer vision methods. These methods focus on searching for points of interest in an image called keypoints, from which a descriptor vector, i.e., the list of features relative to each image (324 features per image) was calculated. The first method applied was SIFT (24), by means of which 164 features were extracted from each image. SIFT returned keypoints on the 12 leads of the ECGs with different orientations and amplitudes along the waveforms of each lead, identifying known ECG-graphic features, such as the R-wave and S-wave peak points of the QRS complex. The second algorithm was BoVW (24), which is designed to detect more complex structures in an image and their occurrence frequency rather than focus on points of the image (25). From the application of BoVW, 160 visual features were extracted per image. This method made it possible to detect larger ECG-graphic features such as the T-wave or the QRS complex. The extracted visual features were subsequently used as input to train a machine learning classification algorithm as numerical variables for the prediction of the neurological outcome of patients at the discharge. Alongside features visually extracted from the ECG images, patient demographic data (age, sex), first rhythm (shockable or not), and ROSC-ECG time (time interval between achieving ROSC and the first 12-lead ECG acquisition) were used as model inputs. Age and sex were included in the model because they are known to influence ECG characteristics (26), and the interval between ROSC to ECG acquisition was incorporated given its potential impact on ECG (27). Presenting rhythm was also included in the model to discern between the two principal categories of outcomes. The ECG-specific features, engineered to enhance model interpretability, are detailed in Supplementary Table S3.

The dataset was partitioned into training, validation, and test subsets using a two-step stratified random split based on neurological outcome with a fixed seed (random_state = 42). First, patients were split into training (80%) and a temporary set (20%); the temporary set was then split equally into validation (10%) and test (10%), ensuring that each patient contributed to only one subset. The validation subset was used for model selection and early stopping to prevent overfitting during training, while the test subset was held out and used only for final performance evaluation.

Baseline demographic and key clinical characteristics (age, sex, ROSC-ECG time, presenting rhythm, and outcome distribution) are reported for the training, validation, and test groups in Table 1. Continuous variables are reported as median [IQR], which represent the 50th percentile and the 25th–75th percentile range, respectively, and provide a robust summary of central tendency and dispersion. We did not stratify the dataset by presenting ECG rhythm to avoid introducing rhythm-specific outcome information into the data split, which could inflate model performance and reduce generalizability. Instead, the natural distribution of presenting rhythms was preserved across groups, allowing the model to be evaluated under conditions that more closely reflect real-world clinical deployment. ROSC-ECG time (ROSC_ECG_TIME) was included as a continuous variable. Post-ROSC ECGs and associated data from any given patient were not included in multiple datasets.

**Table 1:** Baseline characteristics of the training, validation, and test datasets.

Characteristic	Training (*n* = 820)	Validation (*n* = 78)	Test (*n* = 78)
Neurological outcome at discharge, *n* (%)			
Bad (CPC > 2)	545 (66.5)	53 (67.9)	43 (55.1)
Good (CPC ≤ 2)	275 (33.5)	25 (32.1)	35 (44.9)
Age (IQR), years	70 (60–79)	73 (65–81)	68.0 (58–77)
Sex, *n* (%)			
Male	532 (64.9)	43 (55.1)	57 (73.1)
Female	288 (35.1)	35 (44.9)	21 (26.9)
First recorded rhythm, *n* (%)			
Non-shockable	436 (53.2)	48 (61.5)	44 (56.4)
Shockable	377 (46.0)	30 (38.5)	34 (43.6)
ROSC-to-ECG time (IQR), min	7.0 (3.6–15.0)	5.0 (3.0–12.1)	7.2 (4.8–12.0)

**Figure 1:**
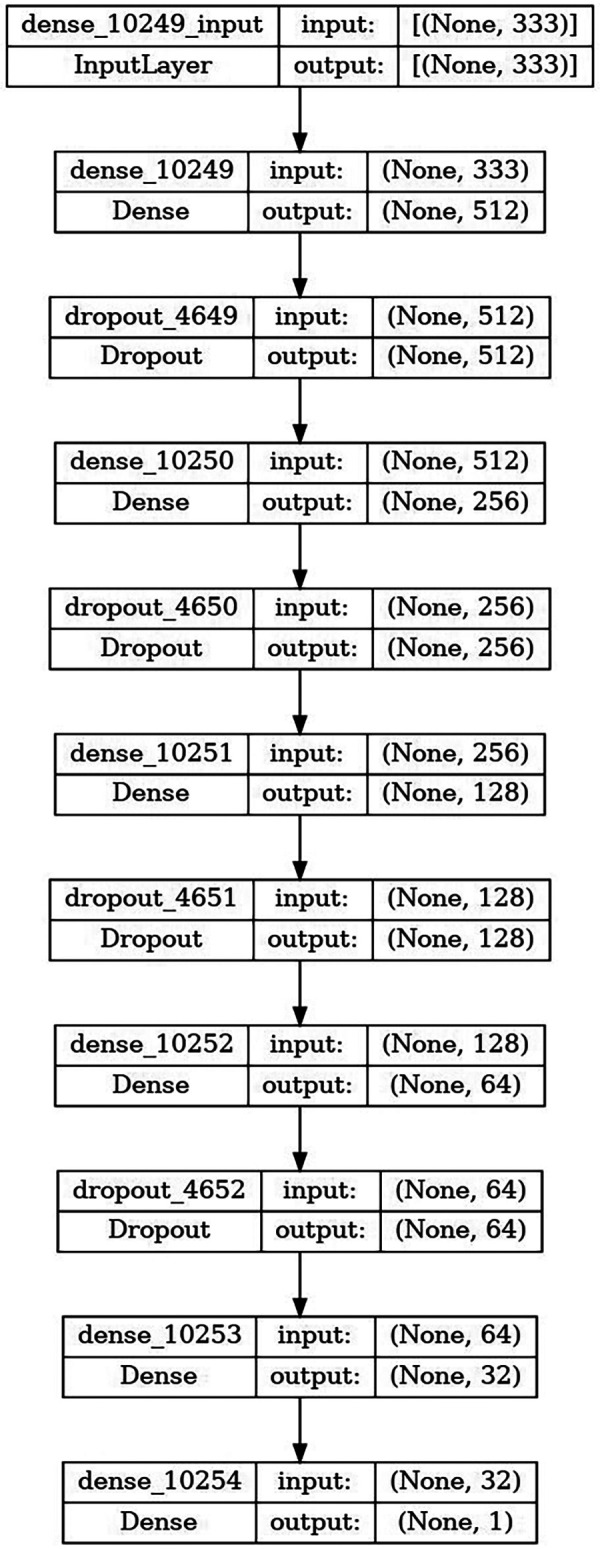
Architecture of the custom deep neural network model consisting of sequential dense and dropout layers with decreasing units for clinical prediction tasks.

A custom deep neural network model (DNN) was developed for the classification task. The architecture comprises an input layer followed by six fully connected (dense) layers with progressively decreasing units (512 → 256 → 128 → 64 → 32 → 1), each interleaved with dropout layers to mitigate overfitting (Figure 1). All hidden layers employed Rectified Linear Unit (ReLU) activation functions, and the model was trained for up to 325 epochs using the Adaptive Moment Estimation (ADAM) (28) optimizer with an initial learning rate of 0.001. To improve generalization, early stopping (patience = 10) was applied, terminating training if the validation performance did not improve for 10 consecutive epochs. Additionally, a learning rate reduction strategy was adopted, whereby the learning rate was automatically decreased if the validation loss plateaued, allowing the model to converge more effectively. The model showed steady improvement during training over 300 epochs, with increasing training and validation accuracy and decreasing loss curves.

To quantify the incremental predictive value of ECG-derived features beyond established clinical predictors, we evaluated three models using the same train/validation/test split (random_state=42): (1) a clinical-only model including age, sex, presenting rhythm, and ROSC-ECG time; (2) an ECG-only model using ECG-derived descriptors without clinical variables; and (3) a combined model including both clinical and ECG-derived features. Performance was reported on the held-out test set. Incremental value of ECG-derived features was quantified as the difference in AUROC, Matthew's correlation coefficient (MCC), accuracy, and balanced accuracy between the combined model and the clinical-only baseline (Supplementary Table S4 and Table S5). Model evaluation was based on accuracy, sensitivity, specificity, positive predictive value (PPV), negative predictive value (NPV), F1-score, AUROC, and MCC. To improve interpretability of the deep learning model, we used SHapley Additive exPlanations (SHAP) (29) a *post hoc* explainability method that quantifies how much each input feature contributes to an individual prediction. A positive SHAP value indicates that a feature pushes the model toward predicting poor neurological outcome (Class 1), whereas a negative SHAP value indicates a contribution toward good neurological outcome (Class 0). The magnitude of the SHAP value reflects the strength of that feature's influence on the model output. Performance on the test set was evaluated using the same classification metrics to ensure comparability (Table 2). To assess calibration, a calibration plot and the Brier score (i.e., the mean squared difference between predicted and observed outcomes) were computed.

**Table 2:** Discriminative metrics calculated on the test dataset for the DNN model score.

Metric	Test dataset
True Positives (TP)	37
False Positives (FP)	9
True Negatives (TN)	26
False Negatives (FN)	6
Accuracy (%)	80.77
Balanced Accuracy (%)	80.17
Sensitivity/Recall (%)	86.05
Specificity (%)	74.29
Precision/PPV (%)	80.44
Negative Predictive Value (%)	81.25
False Positive Rate (%)	25.71
False Discovery Rate (%)	19.57
False Negative Rate (%)	13.95
F1 score (%)	83.15
ROC–AUC	86.00
Matthews Correlation Coefficient	0.61

## 3 Results

### 3.1 Study population

**Figure 2:**
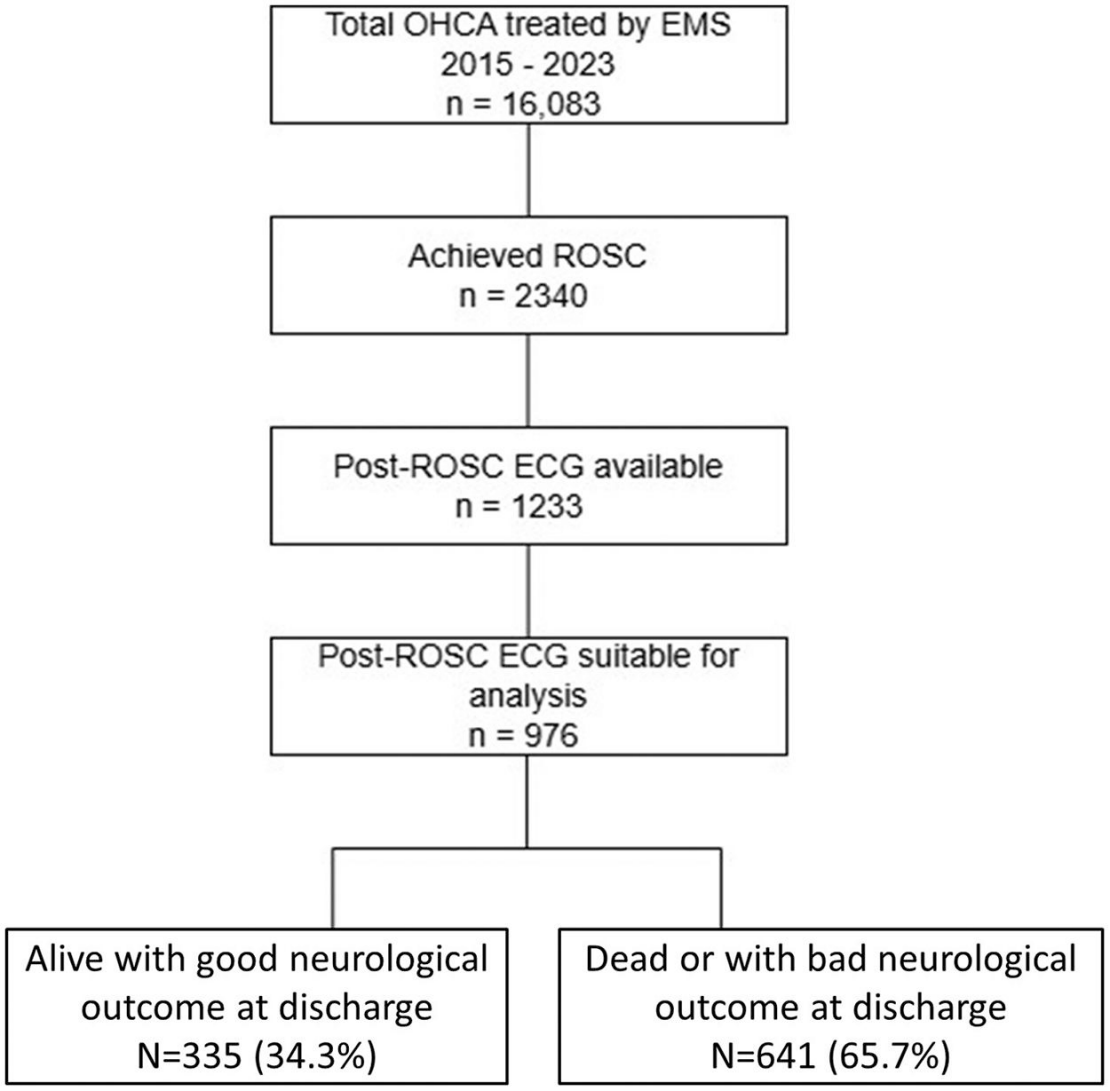
Study flow diagram.

Out of the 16,083 OHCA victims who received resuscitation attempts during the study period, 2,340 achieved ROSC. Among these, the post-ROSC ECG was retrievable in 1,233 cases, with 976 suitable for analysis. At discharge, 641 patients either died or had an unfavorable neurological outcome, while 335 patients were alive with a good neurological outcome (Figure 2).

### 3.2 OHCA characteristics

Patients were mainly males (64.8%) with a median age of 70 years (IQR: 60–79). The majority of OHCAs had presumed medical etiology (94.5%), occurred at home (79.1%) and were witnessed, either by bystanders (59.5%) or EMS personnel (24.7%). The initial rhythm was shockable in 46.2% of cases and CPR was initiated by a bystander in 41.3% of cases. Further details are presented in Table 3.

**Table 3:** Baseline patient and OHCA characteristics.

OHCA characteristics	Total	Poor Outcome	Good Outcome	*p*-value
	*N* = 976	*N* = 641	*N* = 335	
Sex (%)				<0.001
Male	632 (64.8)	375 (58.5)	257 (76.7)	
Female	344 (35.2)	266 (41.5)	78 (23.3)	
Age (IQR), year	70.0 (60.0–79.0)	73.0 (63.0–81.0)	64.0 (56.0–74.0)	<0.001
Etiology (%)				0.008
Medical	922 (94.5)	593 (92.5)	329 (98.2)	
Asphyxia	30 (3.1)	28 (4.4)	2 (0.6)	
Traumatic	16 (1.6)	14 (2.2)	2 (0.6)	
Overdose	5 (0.5)	4 (0.6)	1 (0.3)	
Drowning	2 (0.2)	1 (0.2)	1 (0.3)	
Unknown	1 (0.1)	1 (0.2)	0 (0.0)	
OHCA location (%)				<0.001
Home	772 (79.1)	537 (83.8)	235 (70.1)	
Street	90 (9.2)	51 (8.0)	39 (11.6)	
Public building	28 (2.9)	10 (1.6)	18 (5.4)	
Other	25 (2.6)	13 (2.0)	12 (3.6)	
Nursing home	24 (2.5)	19 (3.0)	5 (1.5)	
Sport	21 (2.2)	3 (0.5)	18 (5.4)	
Workplace	15 (1.5)	8 (1.2)	7 (2.1)	
School	1 (0.1)	0 (0.0)	1 (0.3)	
Witnessed event (%)				<0.001
Yes (bystander)	581 (59.5)	401 (62.6)	180 (53.7)	
Yes (EMS)	241 (24.7)	107 (16.7)	134 (40.0)	
No	133 (13.6)	118 (18.4)	15 (4.5)	
Unknown	21 (2.2)	15 (2.3)	6 (1.8)	
Bystander CPR (%)				0.47
No	573 (58.7)	371 (57.9)	202 (60.3)	
Yes	403 (41.3)	270 (42.1)	133 (39.7)	
First rhythm (%)				<0.001
Non shockable	518 (53.1)	457 (71.3)	61 (18.2)	
Shockable	451 (46.2)	180 (28.1)	271 (80.9)	
Unknown	7 (0.7)	4 (0.6)	3 (0.9)	
Survived to discharge (%)				<0.001
No	584 (59.8)	584 (91.1)	0 (0.0)	
Yes	392 (40.2)	57 (8.9)	335 (100.0)	
ROSC-ECG time (IQR),min^a^	7.0 (3.5–15.0)	6.0 (3.0–13.0)	8.0 (4.9–20.0)	<0.001

IQR, interquartile range; OHCA, Out-of-hospital cardiac arrest; CPR, Cardiopulmonary Resuscitation.

a Time interval between ROSC achievement and first 12-lead ECG acquisition.

### 3.3 Performance of the validation and test datasets

**Figure 3:**
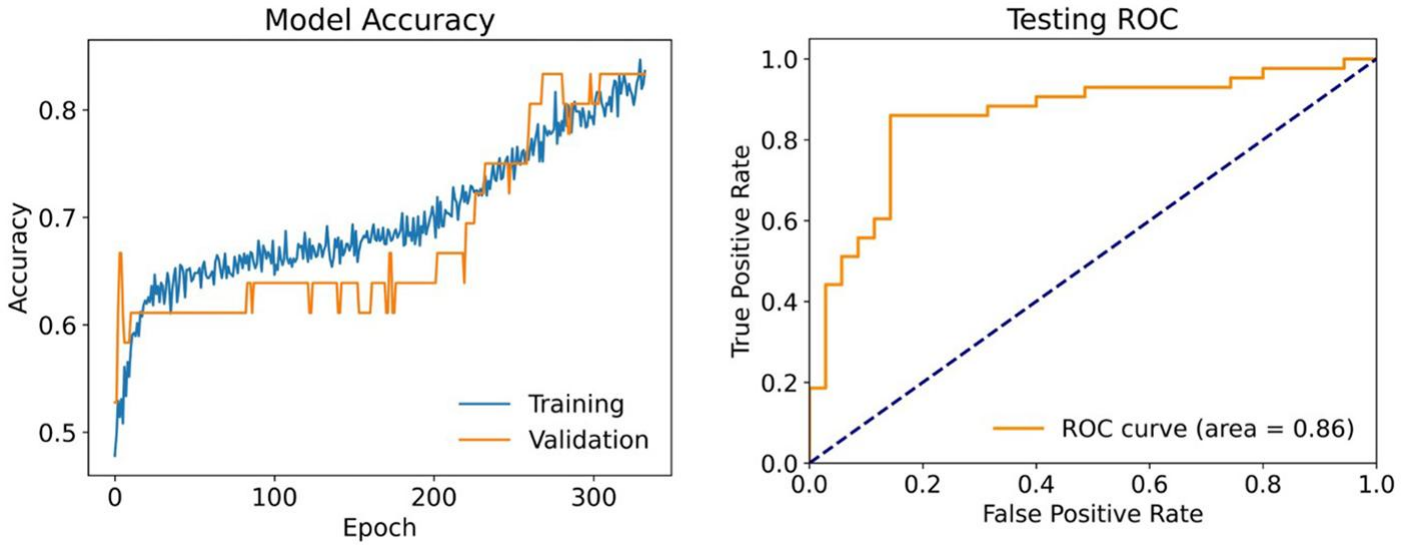
Performance of the DNN model showing increasing training and validation accuracy over epochs (left) and a test AUROC of 0.86 indicating good discriminative ability (right).

**Figure 4:**
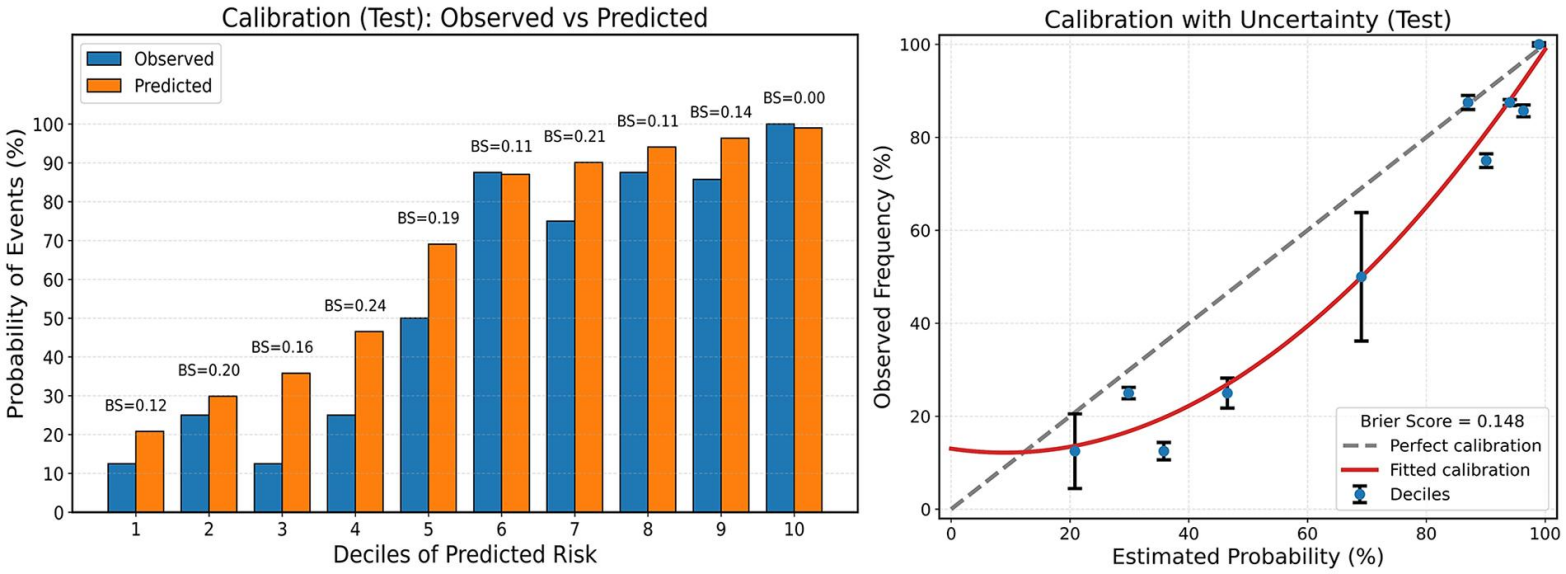
Model calibration performances: the decile stratification plot (left) demonstrates good alignment between observed and predicted risks across risk groups, while the calibration plot (right) indicates overall reliable probability estimates with minor deviations from perfect calibration.

On the independent test set, our DNN model achieved an accuracy of 80.8%, sensitivity 86.1%, specificity 74.3%, PPV of 80.4%, NPV 81.3% and F1-score of 83.1%. The true positive rate was 86%, and the true negative rate was 74.3%. The false negative rate and false positive rate were 13.9% and 25.7%, respectively, while the false discovery rate was 19.6%. The Matthew's correlation coefficient was 0.61 (Table 2). The ROC curve showed strong discriminatory ability with an AUC of 0.86 (Figure 3) with good calibration performance (Figure 4).

In incremental value analyses (Supplementary Tables S4 and S5), the clinical-only model achieved AUC 0.80, while the ECG-only model achieved AUC 0.77. Consistent with the central motivation of this work, integrating ECG-derived features with clinical variables yielded a clear improvement in discrimination and overall classification performance, with absolute gain of *Δ*AUC +0.0563 compared to the clinical-only model, supporting that ECG-derived features provide complementary prognostic information beyond clinical predictors.

**Figure 5:**
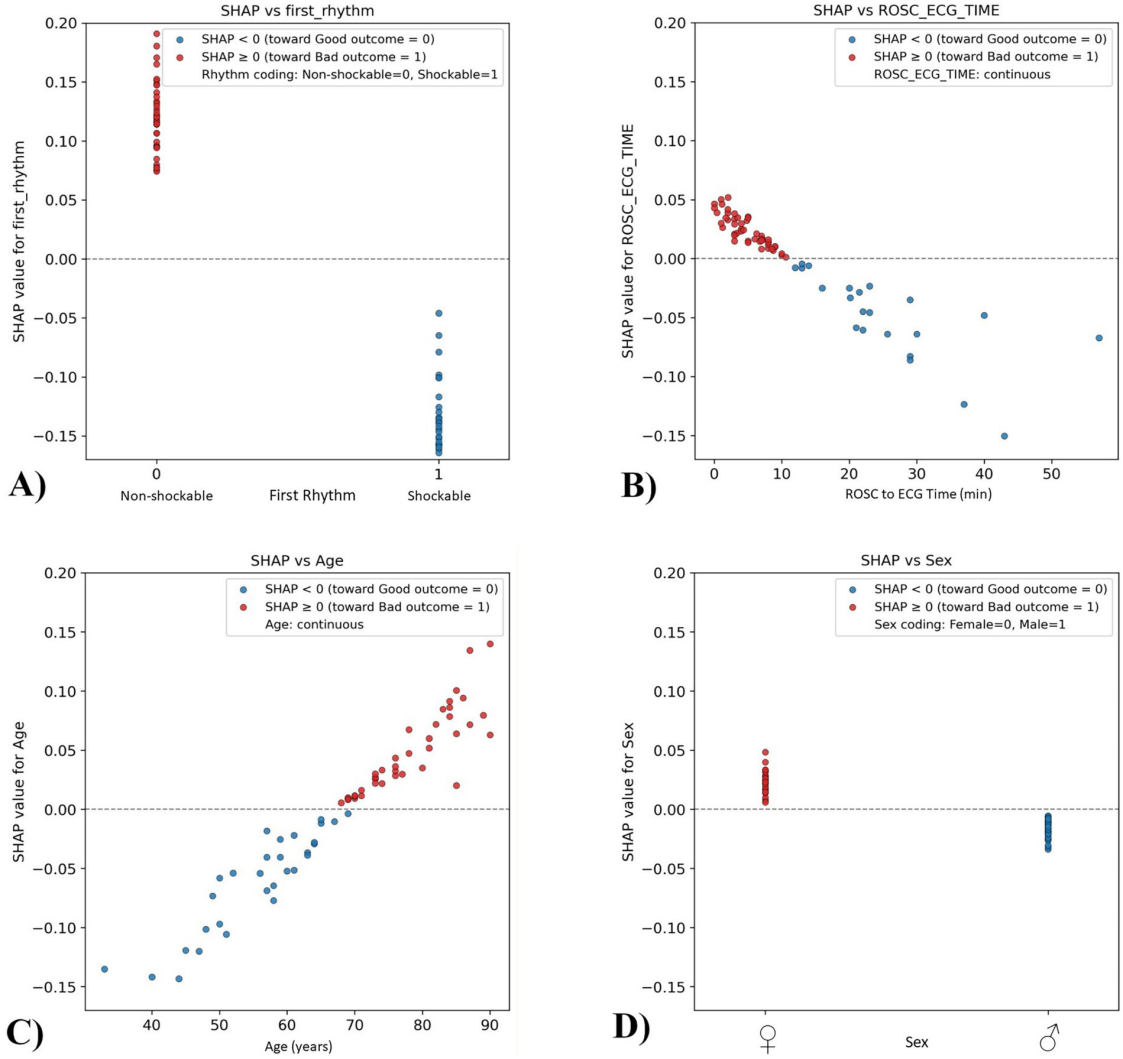
SHAP scatter plots illustrate the influence of **(A)** first rhythm (the first recorded rhythm during OHCA), **(B)** ROSC-ECG time (time between achieving ROSC and the first 12-lead ECG acquisition), **(C)** age (patient age at OHCA), and **(D)** sex (biological sex), on the model's predictions of neurological outcomes.

The SHAP analysis provided meaningful and interpretable insights into the model's decision-making, as illustrated in Figure 5 and Supplementary Figure S2. Analysis of ROSC-ECG time showed that the earlier the acquisition the higher the chance to exhibit features associated with poor neurological outcome. The presenting rhythm was the most influential predictor: the presence of shockable rhythm strongly favored classification into the good neurological outcome group, while non-shockable rhythm supported prediction of poor neurological outcomes. The model also demonstrated clear age dependency: the younger the age the higher the probability to be associated with good neurological outcomes. This age-related trend aligns with clinical expectations in post-OHCA recovery. Regarding sex, the model indicated that males were more often associated with good neurological outcome predictions, whereas females contributed more frequently to poor outcome predictions, potentially reflecting underlying physiological or treatment-response differences.

From the top-ranked SHAP features, we projected the model's attention back onto the ECG waveforms, as shown in Supplementary Figure S3. This analysis step, based on top 10 computer vision features, revealed that the ST-segment consistently exhibited the highest importance in model predictions, followed by the R peak. In later ranking of the SHAP, visual features attending to the QRS complex also contributed, uncovering subtle morphological patterns that the model learned to associate with neurological outcome status. These explainable AI findings reinforce the clinical plausibility of our model's predictions and enhance its utility as a decision-support tool by offering transparent rationale behind each classification.

## 4 Discussion

To the best of our knowledge, this study presents one of the first machine learning approaches leveraging post-ROSC 12-lead ECG data to evaluate the association between ECG-derived features and neurological outcome immediately after ROSC. We have developed and internally validated a machine-learning algorithm intended to support the assessment of neurological outcome at hospital discharge by using post-ROSC 12-lead ECG together with few other variables in patients resuscitated from OHCA. This model demonstrates that integrating ECG-derived features may improve early risk stratification at the scene, prior to hospital admission. Besides its performance, a prominent distinction of our tool is its reliance on patient's individual data, namely the post-ROSC ECG, which is readily obtainable by emergency personnel and recommended by both European and American guidelines (11, 12) for diagnostic purposes. Further external validation is warranted to clarify its generalizability and clinical utility.

### 4.1 The role of post-ROSC ECG

The acquisition of a 12-lead ECG after ROSC is recommended in the pre-hospital setting and drives the decision-making process regarding the need for an emergent coronary angiography (11, 12). It is also a highly significant source of additional information reflecting the patient's condition after ROSC that can help clinicians to triage patients. In a previous work (30), we demonstrated the association between low perfusion after ROSC and survival and we explored the link between the post-ROSC ECG findings and systemic perfusion (31). Specifically, we found that severely reduced peripheral perfusion can be reflected by ECG alterations. This supports the idea that the post-ROSC ECG can predict survival and we proposed a score based on ECG features routinely evaluated by cardiologists (20). That score was able to stratify patients into three risk classes for mortality with a rather good performance (Harrell's c of 0.66). On this basis, we aimed to improve pre-hospital prognostic stratification via artificial intelligence in this study**.**

It is important to highlight that in both the previously developed score (20) and the current DNN model, the S-T wave segment and R peak demonstrated high importance for model prediction (Supplementary Figure S3). These ECG features represent the most informative components in cardiac arrest setting and they are clinically associated with post-ischemic changes. SHAP analysis (Supplementary Figure S2) revealed that those regions had high-impact on model interpretation. Particularly, it is not surprising, and it is worth discussing, that the ROSC to ECG time was associated with outcome. Clearly, it is not a patient specific variable, but it is known that the ST segment may change over time after ROSC. Early detected ST segment elevation may be due to systemic hypoxia or hypo perfusion (31) rather than to a coronary occlusion. It means that even in the presence of ST segment elevation detected immediately after ROSC the cause may be other than STEMI with a resulting lower survival (32). This pattern may also reflect the influence of physiological instability during early acquisition, while delayed acquisition may occur after partial clinical stabilization. In addition, later ECG acquisition may inherently select for patients in relatively more favorable clinical condition, as individuals with more severe instability may deteriorate or die before a later ECG can be obtained.

With our ML model, prognostic performance was enhanced and the need for cardiological interpretation prior to hospital admission was reduced.

### 4.2 Performance and advantages

The robustness of our DNN model is demonstrated by its effectiveness when applied to ECGs collected from a large geographic area encompassing more than 30 Hospitals. Our model can be applied without any limitations with regards to equipment because the tool has been fed by ECGs retrieved from different monitors/defibrillators. This tool does not need trained personnel, being easy to use and cost-effective. Since we included all causes of cardiac arrest - with medical etiology being by far the most common, as expected—our model can be used widely and easily for all cardiac arrest cases. Moreover, it is not jeopardized by inter-observer subjectivity, thus representing a possible form of standardization. Because of the intrinsic nature of machine learning, the model is flexible and improvable. This means that the performance of the model, already good at present, may further improve by feeding the ML model with a higher number of post-ROSC ECGs.

### 4.3 Comparison with existing predictors of survival at discharge after OHCA

The prediction of survival after cardiac arrest is one of the most challenging, and consequently fascinating fields in medicine, especially in the pre-hospital setting. Prognostication can serve different purposes depending on when it is performed. It may occur before ACLS/ALS is initiated (9, 10), to estimate the chance of sustained ROSC, potentially guiding the decision about undertaking resuscitation; during ACLS/ALS, to help guide the resuscitation efforts (33, 34); after ROSC on scene, to direct patients at higher risk of death to the most appropriate and equipped hospital (8); or, finally, after hospital admission to support treatment withdrawal to prevent futile care (4–7). In the pre-hospital setting, the primary goal is to keep patients alive and to increase their chances of survival by choosing the most appropriate treatments and triage based on their clinical condition. This approach is in accordance with our model. It is, therefore, not surprising that our model slightly overestimates the risk of death or of poor neurological outcome at discharge. In the pre-hospital setting, a slight risk overestimation is preferable to prevent rescuers from depriving patients of adequate advanced care. This is different from the multi-parametric approach made 48–72 h after ICU admission, when decisions regarding treatment withdrawal must be made by physicians if unfortunate outcomes are deemed inevitable (3). In this context, models that underestimate the risk of poor outcome are preferable to reduce the likelihood of inappropriately withholding care.

Literature provides some in-hospital scores which aimed to predict neurological outcome at discharge such as the OHCA score (7), the CAHP score (5), the TTM score (6) and the MIRACLE_2_ score (4) (Supplementary Table S6). Besides their different settings of applicability (pre-hospital vs. in-hospital) and consequently their distinct primary goals, our model is the first to investigate neurological outcome in the pre-hospital setting based on post-ROSC ECGs without relying on traditional statistical methods. The OHCA score by Adrie et al., created by using 130 adult OHCA patients admitted to ICU and validated on a cohort of 210 patients, showed a good performance in predicting death or severe neurological impairment (CPC >2) at discharge with an AUC 0.82 (95% CI: 0.70–0.95). However, the variables included in this score were lactate and creatinine, which are not available in a pre-hospital setting (7). Similarly, the CAHP score developed in Paris by Maupan et al. used arterial pH, which is far less frequently available compared to the post-ROSC ECG (5). The TTM score by Martinell and the MiRACLE_2_ by Pareek et al. predicted neurological outcome at 6 months by using non-reactive pupils and arterial pH (4, 6). While all these scores have acceptable predictive accuracy, their exportability to the pre-hospital setting is unfeasible.

The only existing pre-hospital score that was developed with the aim of predicting survival at discharge is the ACLS score (8), published in JAMA by Eisenberg et al. more than 40 years ago by using 611 OHCA patients from suburban Seattle, USA. As with other pre-hospital scores (9, 10), the ACLS score included variables which were not patient-centered, but focused on OHCA circumstances such as witnessed status of cardiac arrest, presenting rhythm, bystander initiating cardiopulmonary resuscitation (CPR), paramedic response time. Additionally, this score failed the external validation (35). In contrast, our DNN model offers multiple advantages: (1) it is associated with survival at hospital discharge and good neurological outcome; (2) it outperforms the ACLS score by reaching an AUROC of 0.86 in the test set (Figure 3); (3) it relies on data directly acquired from the patient, the post-ROSC ECG, which also serves as a surrogate of systemic perfusion of the patient. While OHCA characteristics are undoubtedly important with regards to patients' prognoses, basing risk scores solely on these factors have the potential to level the risk of vastly different patients. On the contrary, the 12-lead ECG enables a more individualized risk assessment for each resuscitated patient.

### 4.4 Limitations

This study has some limitations. (1) It is a retrospective evaluation of post-ROSC ECGs based on an Italian cohort. Even though we provided a validation set on post-ROSC ECGs not seen by the model, a validation on a larger cohort with ECGs from other countries as well as a prospective study are needed. (2) The model does not incorporate circumstantial factors of the cardiac arrest such as location, witnessed status at presentation, no/low flow time, administered drugs during ACLS, and the application of mechanical chest compression device. This approach was adopted intentionally in order to give appropriate weight to the post-ROSC ECG for the prediction of neurological outcome. Once established the association between neurological outcome and post-ROSC ECG, further studies will incorporate additional Utstein variables. We anticipate that integrating key arrest-related factors, such as arrest-to-ROSC time, drug administration, and mechanical chest compression use, alongside post-ROSC ECG features will further enhance neurological outcome prediction and improve overall model performance. (3) Despite excluding suboptimal ECGs, some may still have been misclassified by the model due to artifacts or missing data in the pdf format. We believe that by increasing the number of ECGs we will reduce the rate of potential misclassification. (4) Since this study was conducted within a specific regional EMS organization (Lombardy, Italy), the generalizability of the tool may vary depending on local EMS strategies (e.g., stay-and-play vs. rapid transport with ongoing CPR). Variations in crew composition, protocols, and documentation practices across international EMS systems could influence both the feasibility of implementing the model and its prognostic performance. Future steps include external validation to assess the international applicability of the model, ideally in a prospective manner. (5) Since ECGs were analyzed from digital documents (PDF/JPEG) using image-based descriptors, variability in export resolution, compression, and grid/background quality may have influenced feature stability despite fixed 300-DPI conversion and preprocessing. (6) Although the training/validation/test split was stratified by outcome, it was not stratified by other variables (e.g., presenting rhythm) and was performed within the same dataset; therefore, residual distributional differences across subsets and potential cohort-specific patterns may lead to optimistic internal estimates. We mitigated this by reporting balanced accuracy/MCC and providing subset-level baseline characteristics (Tables 1, 2), and future work will include repeated cross-validation and prospective external validation.

## 5 Conclusion

To the best of our knowledge, this is the first machine-learning tool based on post-ROSC 12-lead ECGs designed to contribute to the assessment of neurological outcome in the pre-hospital setting. Our DNN model is intended as an easily applicable tool that may support early stratification of patients' prognosis after an out-of-hospital cardiac arrest, therefore supporting EMS personnel in their decision-making process about ACLS treatment, triage and transport. Further external validation is required to determine the generalizability and clinical utility of this tool across diverse healthcare systems, particularly in light of variability in EMS organization and practice worldwide.

## Data Availability

The raw data supporting the conclusions of this article will be made available by the authors, without undue reservation.

## Supplementary material

Supplementary Figure S1 Examples of common ECG signal-quality failure modes in the dataset: (A) noise/artifacts (e.g., motion or CPR artifact, baseline wander, electrical interference); (B) preprocessing/digitization failure (e.g., grid-cleaning leading to waveform loss or partial deletion); (C) lead-quality issues (e.g., missing or intermittent leads due to poor contact).

Supplementary Figure S2 SHAP summary plot showing the most influential features on the test set.

Supplementary Figure S3 Visualization of the top two image-based features contributing to model predictions as identified by SHAP analysis. The left panel highlights keypoint activations corresponding to sift_angle_24, and the right panel shows sift_angle_22, both overlaid on cleaned ECG traces. These markers represent high-impact regions for model interpretation, as determined from the SHAP summary plot.
