# Supplementary material for: Artificial intelligence applied to post-resuscitation ECGs for early prognostication after out-of-hospital cardiac arrest
Source: Front Cardiovasc Med. 2026 May 11;13:1765751. doi: 10.3389/fcvm.2026.1765751 (PMC13199275; doi:10.3389/fcvm.2026.1765751)
Supplement: Supplementary Figure S1 — Examples of common ECG signal-quality failure modes in the dataset: (A) noise/artifacts (e.g., motion or CPR artifact, baseline wander, electrical interference); (B) preprocessing/digitization failure (e.g., grid-cleaning leading to waveform loss or partial deletion); (C) lead-quality issues (e.g., missing or intermittent leads due to poor contact). [file Datasheet1.docx]

**SUPPLEMENTARY MATERIALS**

**Supplementary Materials and Methods**

- Setting and Emergency Medical System (EMS) Description
- Lombardia Cardiac Arrest Registry (Lombardia CARe) Description
- ECG files preprocessing
- Software and libraries

**Supplementary tables**

- Table S1. Characteristics collected in the OHCA registry according to Utstein recommendations
- Table S2. Reasons for exclusion of sub-optimal post-ROSC ECGs (n = 257 excluded)
- Table S3. Features used for model training
- Table S4. Predictive performance comparison of the clinical-only baseline, ECG-only model, and the final combined DNN, reporting accuracy (ACC), balanced accuracy (BAAC), MCC, ROC-AUC, and the incremental gain of adding ECG-derived features beyond clinical variables (test set; random_state = 42), including statistical tests.
- Table S5. Absolute performance differences (ΔAUC, ΔACC, ΔBACC, ΔMCC) comparing Vision vs Clinical on the fixed test set and Combined vs Clinical/Vision across the article and fixed test splits.
- Table S6.Comparison of characteristics between the existing scores in out-of-hospital cardiac arrest.

**Supplementary figures**

- Figure S1. Examples of common ECG signal-quality failure modes in the dataset: (A) noise/artifacts (e.g., motion or CPR artifact, baseline wander, electrical interference); (B) preprocessing/digitization failure (e.g., grid-cleaning leading to waveform loss or partial deletion); (C) lead-quality issues (e.g., missing or intermittent leads due to poor contact or motion).
- Figure S2. SHAP summary plot showing the most influential features on the test set.
- Figure S3. Visualization of the top two image-based features contributing to model predictions as identified by SHAP analysis.

**Supplementary Materials and Methods**

**Setting and Emergency Medical System (EMS) Description**

The territory covered by the LombardiaCARe encompasses an area of 15126 km2, covering a population of 4243.857 millions of inhabitants divided as follows: Pavia 2,969 km2 ; 534,506 inh., Lodi 783 km2 ; 227,327 inh., Cremona 1,770 km2 ; 351,654 inh., Mantua 2,341 km2 ; 404,476 inh., Varese 1,198 km2; 877,668 inh., Como 1,279 km2 ; 594,941 inh. and Brescia 4,786 km2 ; 1.253.157 inh. , as of 1st January 2022.

The Emergency Medical Service is provided by the Agenzia Regionale dell’Emergenza Urgenza (AREU), which covers the whole Lombardy region. There are four different EMS dispatch centers, which coordinate rescues among the provinces, named “Sale Operative Regionali dell’Emergenza Urgenza” (SOREU):

- SOREU della Pianura: EMS dispatch center for the provinces of Pavia (PV), Lodi (LO), Cremona (CR), Mantua (MN) and the western part of Milan (MI) province (this not yet covered by the Registry); it coordinates 48 ambulances staffed with basic life support and defibrillation (BLS-D)-trained personnel, and 22 advanced life support (ALS)-trained staffed vehicles (a physician and a specialized nurse or a specialized nurse only).

- SOREU dei Laghi: EMS dispatch center for the provinces of Como (CO), Lecco (LC), Monza (MB) and Varese (VA); it coordinates 56 ambulances staffed with basic life support and defibrillation (BLS-D)-trained personnel, and 21 advanced life support (ALS)-trained staffed vehicles (a physician and a specialized nurse or a specialized nurse only).

- SOREU delle Alpi: EMS dispatch center for the provinces of Brescia (BS), Bergamo (BG) and Sondrio (SO) (BG and SO are not yet covered by Lombardia CARe); it coordinates 91 ambulances staffed with basic life support and defibrillation (BLS-D)-trained personnel, and 41 advanced life support (ALS)-trained staffed vehicles (a physician and a specialized nurse or a specialized nurse only).

- SOREU Metropolitana: EMS dispatch center for the city of Milan and the eastern part of its province (not involved in the present study).

**Lombardia Cardiac Arrest Registry (Lombardia CARe) Description**

The Lombardia Cardiac Arrest Registry (Lombardia CARe - NCT03197142) is a multicenter longitudinal prospective registry resulting from collaboration between the Fondazione IRCCS Policlinico San Matteo and the Azienda Regionale Emergenza Urgenza (AREU) representing the local emergency medical services (EMS). The registry was initiated in October 2014, at which time it covered only the province of Pavia and was designated the Pavia Cardiac Arrest Registry (Pavia CARe). We then extended enrollment to the provinces of Lodi, Cremona, and Mantova on January 1st, 2019, and changed the name to Lombardia CARe. We added the province of Varese on January 1st, 2020, Como and Brescia in January 2021. The province of Lecco, Bergamo and Sondrio are in the process of being made part of the registry and our ultimate goal is to cover the entire Lombardy Region with its 10,000,000 inhabitants.

The present study includes data from the provinces of Pavia, Lodi, Cremona, Mantova, Varese, Como and Brescia in the Lombardy region of northern Italy. The registry was approved by the ethics committee of the Fondazione IRCCS Policlinico San Matteo (P-20140028219) and by the ethics committee of each of the individual provinces involved. All data are collected following Utstein 2014 recommendations (1) and managed using REDCap electronic data capture tools hosted by the Fondazione IRCCS Policlinico San Matteo. REDCap (Research Electronic Data Capture) is a secure, web-based software platform designed to support data capture for research studies, providing 1) an intuitive interface for validated data capture; 2) audit trails for tracking data manipulation and export procedures; 3) automated export procedures for seamless data downloads to common statistical packages; and 4) procedures for data integration and interoperability with external sources (2,3).

**ECG files preprocessing**

The aim of preprocessing the files was to isolate only the parts of the ECGs corresponding to the 12-leads and to prepare the files for the extraction of visual features. Thus, a pipeline of image processing methods was developed to homogenize the ECGs acquired from the various types of defibrillators/monitors. The process began by converting the Portable Document Formatted (PDF) files into images through cropping and resizing. Subsequently, the background gridlines were removed by employing a filter for the color and intensity of the lines. Ultimately, each ECG was transformed into a binary image file, represented in black and white format.

**Software and libraries**

The development of the model was performed in the Python programming language, with the help of an open-source library (AutoSklearn) that automates the learning and optimization processes (4).

## **Supplementary tables**

**Table S1.** Characteristics collected in the OHCA registry according to Utstein recommendations.

| Utstein characteristics |
| --- |

| 1. Age |
| --- |
| 1. Gender |
| 1. OHCA date |
| 1. OHCA time |
| 1. Collapse estimated time |
| 1. Call Time |
| 1. EMS arrival time in place |
| 1. EMS arrival time on patient |
| 1. BLS arrival time (ambulance) |
| 1. ALS-1 arrival time (nurse car) |
| 1. ALS-2 arrival time (physician car) |
| 1. First rhythm analysis time |
| 1. CPR start time |
| 1. CPR started by |
| 1. Etiology (Utstein 2014) |
| 1. OHCA Location |
| 1. Telephone CPR |
| 1. Witnessed OHCA |
| 1. Bystander CPR |
| 1. First monitored rhythm |
| 1. Rhythm type |
| 1. AED used before EMS arrival |
| 1. AED shock before EMS arrival |
| 1. First shock date |
| 1. First shock time |
| 1. First shock delivered by |
| 1. Nr. Delivered shocks |
| 1. ACLS protocol started |
| 1. Adrenaline mg administered |
| 1. Amiodarone |
| 1. Chest compression device |
| 1. Device type |
| 1. CPR Feedback device |
| 1. Stop CPR date |
| 1. Stop CPR time |
| 1. Stop CPR cause |
| 1. Sustained ROSC |
| 1. Survived event |
| 1. Hospital |
| 1. Other Hospital |
| 1. Status upon hospital admission |
| 1. Em. Dept arrival date |
| 1. Em. Dept arrival time |
| 1. ROSC at admission |
| 1. Survived to discharge |
| 1. Neurological outcome at hospital discharge |
| 1. STEMI at ECG |
| 1. Death date |
| 1. Death Cause |
| 1. Survival time (days) |
| 1. 1-month survival |
| 1. Neurological outcome at 1-month follow-up |
| 1. 6-months survival |
| 1. Neurological outcome at 6-months follow-up |
| 1. 1-year survival |
| 1. Neurological outcome at 1-year follow-up |

**Table S2.** Reasons for exclusion of sub-optimal post-ROSC ECGs (n = 257 excluded)

| **Exclusion reason** | **n (%)** |
| --- | --- |
| Lead-quality issues (missing/intermittent leads; poor contact/motion) | 87 (33.9) |
| Preprocessing / digitization failure (grid-cleaning caused waveform loss/partial deletion) | 77 (30.0) |
| Noise / artifacts (motion/CPR artifact, baseline wander, electrical interference) | 93 (36.2) |
| **Total excluded** | **257 (100)** |

**Table S3.** Features used for model training

| **Feature Name** | **Description** | **Data Type** | **Values** |
| --- | --- | --- | --- |
| signal_lines | Number of rows of the ECG | Categorical | 3, 6, 12 |
| signal_columns | Number of columns of the ECG | Categorical | 1, 2, 4 |
| signal_broken | Is any of the ECG leads discontinuous? | Categorical | Yes / No |
| missing_or_straight_lead | Is any of the ECG leads missing or flat? | Categorical | Yes / No |
| signal_noise | Is there noise on the ECG signals? | Categorical | Yes / No |
| signal_artifact | Are there any ECG artifacts? | Categorical | Yes / No |
| BoVW (feature 1 – 160) | Features extracted with the Bag of Visual Words method | Numerical, float | Unspecified |
| SIFT (feature 1 – 164) | Features extracted with the SIFT method | Numerical, float | Unspecified |
| Sex | The biological sex of the patient | Categorical | Male/Female |
| Age | The age of the patient | Numerical, integer | 56 – 81 years |
| First rhythm | The presenting rhythm during cardiac arrest | Categorical | Shockable / Non-shockable |
| ROSC-ECG time | The time between ROSC and first 12-lead ECG acquisition | Numeric, integer | 3 – 20 minutes |

**Table S4.** Predictive performance comparison of the clinical-only baseline, ECG-only model, and the final combined DNN, reporting accuracy (ACC), balanced accuracy (BACC), MCC, ROC-AUC, and the incremental gain of adding ECG-derived features beyond clinical variables (test set; random_state = 42), including statistical tests.

| **Model / Comparison** | **AUC** | **ACC** | **BACC** | **MCC** | **Notes** | **Confusion Matrix [[TN, FP], [FN, TP]]** |
| --- | --- | --- | --- | --- | --- | --- |
| Clinical Features | 0.8008 | 0.7692 | 0.7409 | 0.4817 |  | [[32, 3], [18, 25]] |
| Vision Features | 0.7652 | 0.6923 | 0.6731 | 0.3776 |  | [[17, 18], [6, 37]] |
| DeLong: Vision - Clinical | **-0.0356** |  |  |  | p = 0.653;  95% CI [-0.199, +0.117] |  |
| Combined Model | 0.8571 | 0.8077 | 0.8017 | 0.6100 |  | [[26, 9], [6, 37]] |

**Table S5.** Absolute performance differences (ΔAUC, ΔACC, ΔBACC, ΔMCC) comparing Vision vs Clinical on the fixed test set and Combined vs Clinical/Vision across the article and fixed test splits.

| **Comparison** | **ΔAUC** | **ΔACC** | **ΔBACC** | **ΔMCC** |
| --- | --- | --- | --- | --- |
| Vision – Clinical | **−0.0356** | −0.0769 | −0.0678 | −0.1041 |
| Combined - Clinical | **+0.0563** | +0.0385 | +0.0608 | +0.1283 |
| Combined - Vision | **+0.0919** | +0.1154 | +0.1286 | +0.2324 |

**Table S6. Comparison of characteristics between the existing scores in out-of-hospital cardiac arrest.**

| **SCORE** | **SETTING** | **VARIABLES** | **OUTCOME** |
| --- | --- | --- | --- |
| **ROSC after cardiac arrest**  **(RACA) score** (5) **2011** | OHCA circumstantial factors | Variables:   1. gender 2. age 3. witnessed by lay people/professional 4. initial ECG 5. location (MD office/medical institution/ public place/nursing home) 6. bystander CPR 7. etiology 8. EMS arrival time | Return of spontaneous circulation (ROSC) |
| **Utstein-Based (UB) ROSC score** (6) **2020** | OHCA circumstantial factors | Variables:   1. age 2. etiology 3. location 4. witnessed status 5. bystander CPR 6. EMS arrival time 7. shockable rhythm | Sustained ROSC and survival to hospital admission |
| **ACLS* score** (7)  **1981** | OHCA circumstantial factors | Variables:   1. witnessed status 2. cardiac rhythm 3. person initiating CPR 4. paramedic response time | Survival at discharge |
| **OHCA score** (8)  **2006** | Patient characteristics on admission | Variables:   1. initial rhythm 2. no-flow time 3. low-flow time 4. lactate 5. creatinine | Death or poor neurological outcome (CPC >2) at discharge |
| **Cardiac Arrest Hospital Prognosis (CAHP) score** (9) **2016** | Patient characteristics on admission | Variables:   1. age 2. presenting rhythm 3. home setting arrest 4. collapse-BLS time 5. BLS-ROSC time 6. epinephrine dose 7. admission pH | Poor neurological outcome (CPC >2) at ICU discharge |
| **Target Temperature Management (TTM) score** (10)  **2017** | Circumstantial factors and patient characteristics on admission | Variables:   1. age 2. location 3. initial rhythm 4. no flow time 5. low flow time 6. epinephrine use 7. corneal and pupillary reflexes 8. GCS 9. pH on admission 10. PaCO2 on admission | Survival and neurological outcome (CPC) at 6 months after OHCA. |
| **MIRACLE_2_ score** (11) **2020** | Circumstantial factors and patient characteristics on admission | Variables:   1. witnessed status 2. non-shockable rhythm 3. reactivity of pupils 4. age 5. changing intra-arrest rhythms 6. pH 7. epinephrine administration | Poor neurological  outcome (CPC >2) at 6-month follow-up |

*ACLS* acronym: A (arrest witnessed), C (cardiac rhythm), L (lay bystander CPR), R (response time of paramedic unit)*

## **Supplementary Figures**


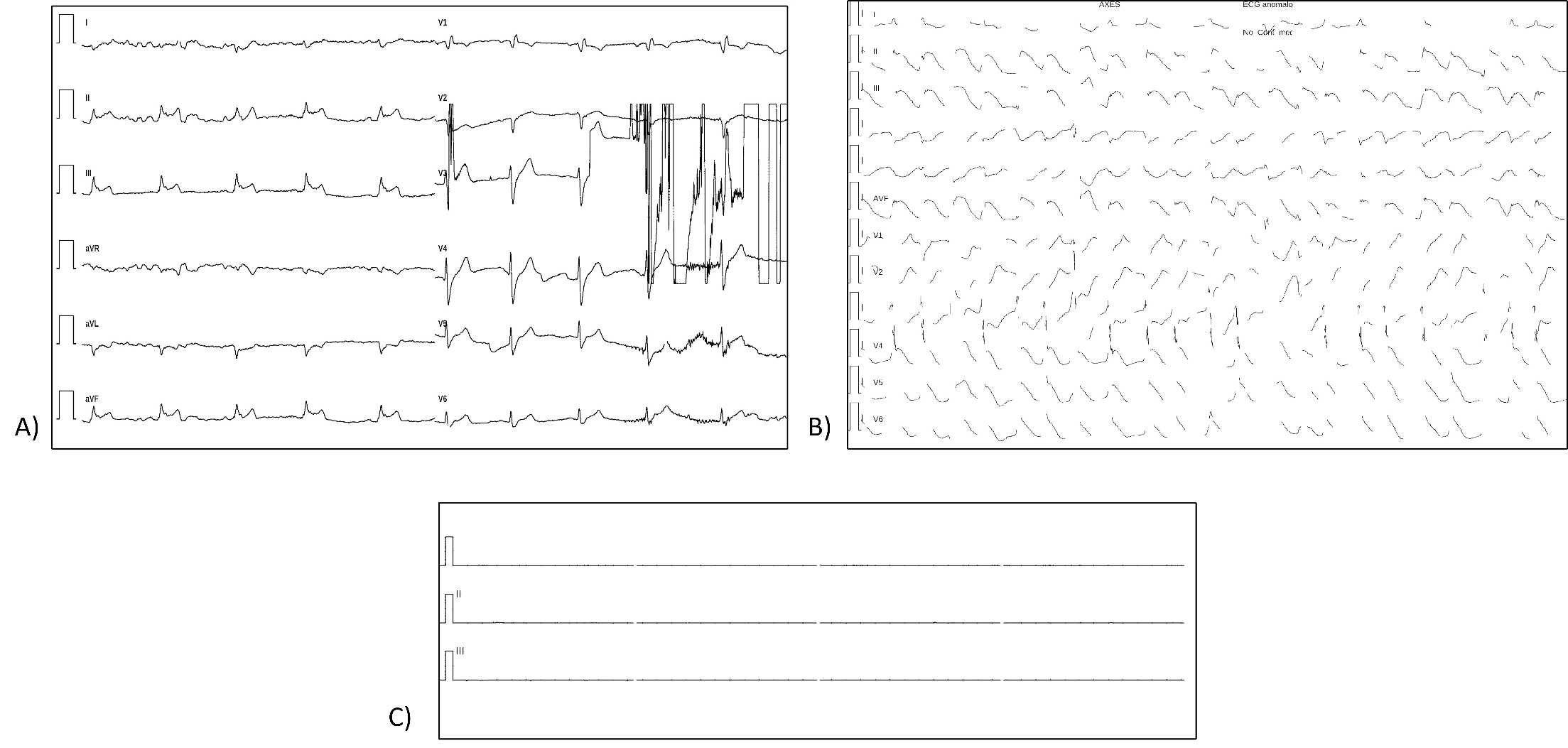


***Figure S1****. Examples of common ECG signal-quality failure modes in the dataset: (A) noise/artifacts (e.g., motion or CPR artifact, baseline wander, electrical interference); (B) preprocessing/digitization failure (e.g., grid-cleaning leading to waveform loss or partial deletion); (C) lead-quality issues (e.g., missing or intermittent leads due to poor contact or motion).*


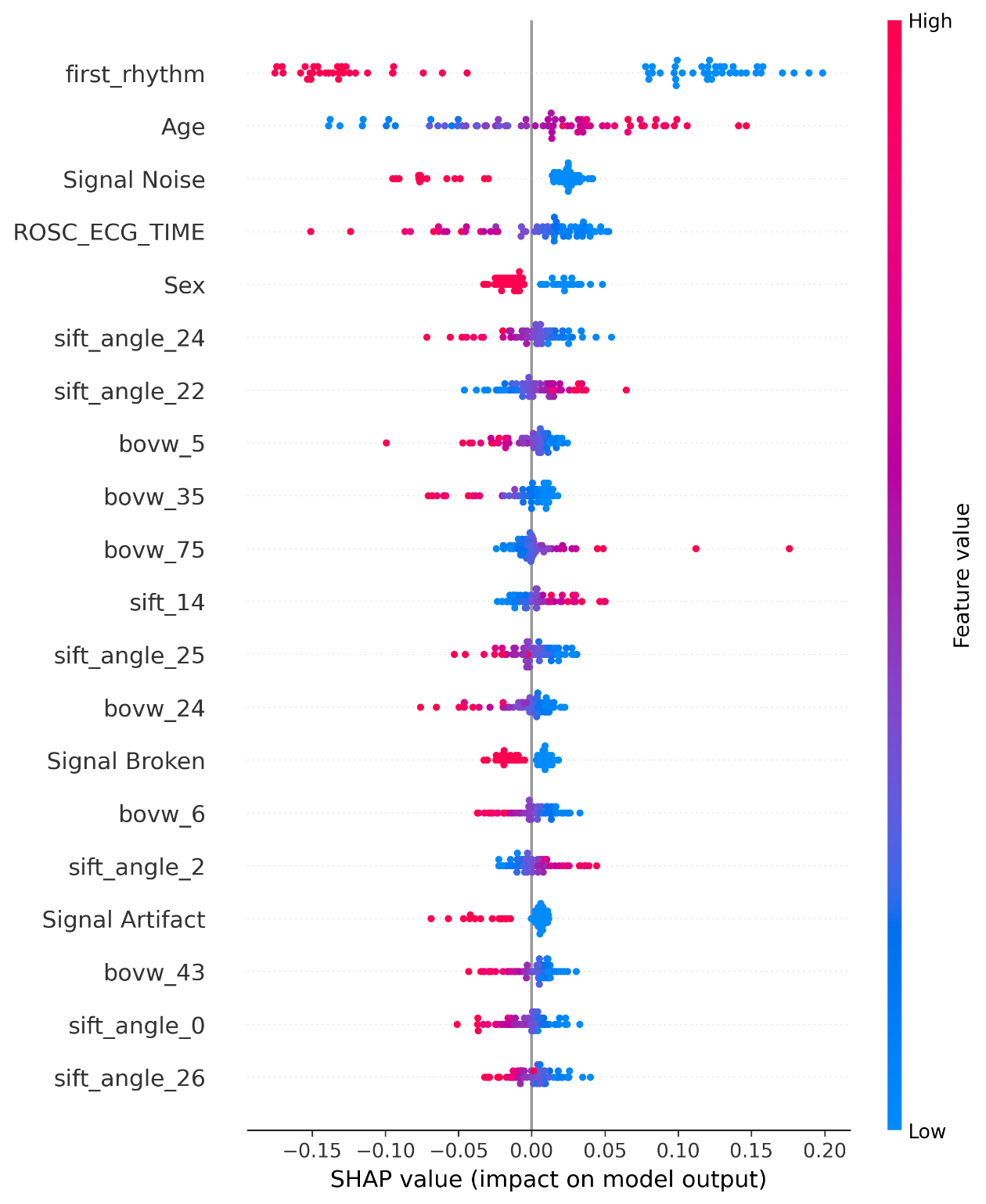


**Figure S2.** SHAP summary plot showing the most influential features on the test set.

With regards to figure S2 above and Figure 5 in the main manuscript, we used SHapley Additive exPlanations (SHAP) to interpret the contribution of each variable to the model predictions. SHAP is based on cooperative game theory and assigns each feature a value representing its contribution to moving a prediction away from the model’s average output. In this study, positive SHAP values indicate a shift toward poor neurological outcome, while negative SHAP values indicate a shift toward good neurological outcome. In the summary plot, features are ranked by overall importance according to the average absolute SHAP value; each point represents one patient, the x-axis shows the direction and magnitude of that feature’s contribution to the prediction, and the color indicates the original feature value from low (blue) to high (red). For binary variables, the color reflects the encoded category. For binary variables, the color separates the two encoded categories.


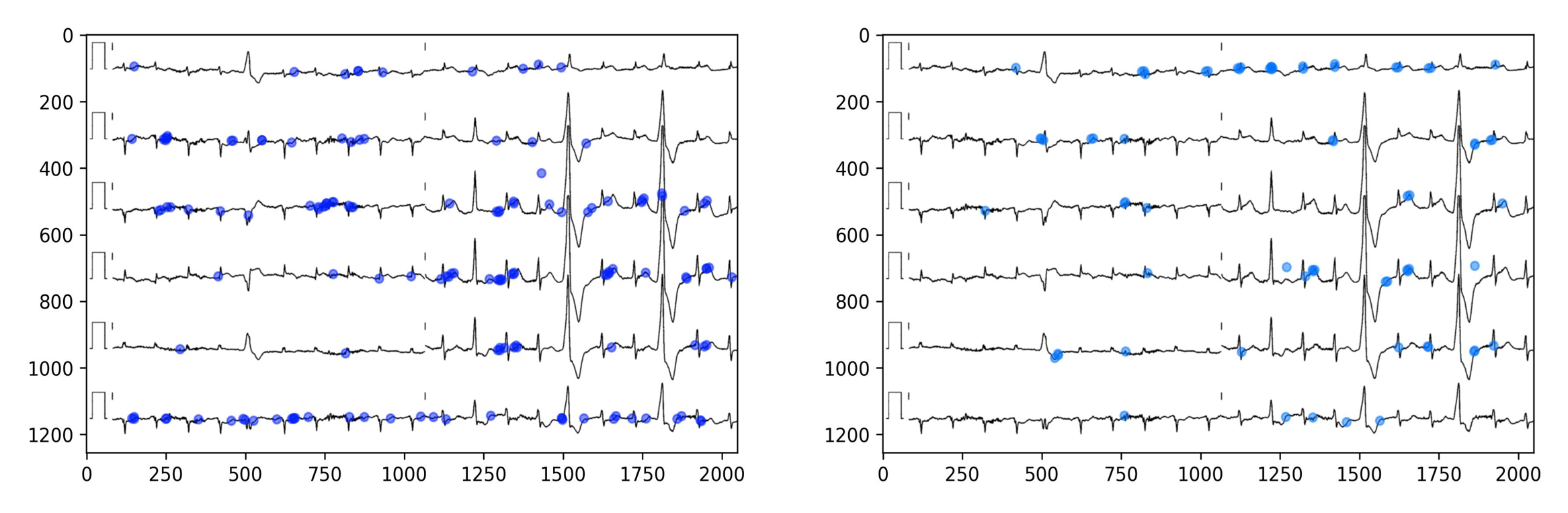


**Figure S3**. Visualization of the top two image-based features contributing to model predictions as identified by SHAP analysis. The left panel highlights keypoint activations corresponding to sift_angle_24, and the right panel shows sift_angle_22, both overlaid on cleaned ECG traces. These markers represent high-impact regions for model interpretation, as determined from the SHAP summary plot.

**References**

1. Perkins GD, Jacobs IG, Nadkarni VM, Berg RA, Bhanji F, Biarent D, et al. Cardiac arrest and cardiopulmonary resuscitation outcome reports: Update of the Utstein resuscitation registry templates for out-of-hospital cardiac arrest: A statement for healthcare professionals from a task force of the international liaison committee . Circulation. 2015;132(13):1286–300.

2. Harris PA, Taylor R, Minor BL, Elliott V, Fernandez M, O’Neal L, et al. The REDCap consortium: Building an international community of software platform partners. J Biomed Inform [Internet]. 2019;95(May):103208. Available from: https://doi.org/10.1016/j.jbi.2019.103208

3. Harris PA, Taylor R, Thielke R, Payne J, Gonzalez N, Conde JG. Research electronic data capture (REDCap)-A metadata-driven methodology and workflow process for providing translational research informatics support. J Biomed Inform. 2009;

4. Feurer M, Eggensperger K, Falkner S, Lindauer M, Hutter F. Auto-Sklearn 2.0: Hands-free AutoML via Meta-Learning. J Mach Learn Res [Internet]. 2022 [cited 2023 Nov 11];23:1–61. Available from: http://jmlr.org/papers/v23/21-0992.html.

5. Gräsner JT, Meybohm P, Lefering R, Wnent J, Bahr J, Messelken M, et al. ROSC after cardiac arrestthe RACA score to predict outcome after out-of-hospital cardiac arrest. Eur Heart J. 2011;32(13):1649–56.

6. Baldi E, Caputo ML, Savastano S, Burkart R, Klersy C, Benvenuti C, et al. An Utstein-based model score to predict survival to hospital admission: The UB-ROSC score. Int J Cardiol [Internet]. 2020;308:84–9. Available from: https://doi.org/10.1016/j.ijcard.2020.01.032

7. Eisenberg M, Hallstrom A, Bergner L. The ACLS Score: Predicting Survival From Out-of-Hospital Cardiac Arrest. JAMA J Am Med Assoc. 1981;246(1):50–2.

8. Adrie C, Cariou A, Mourvillier B, Laurent I, Dabbane H, Hantala F, et al. Predicting survival with good neurological recovery at hospital admission after successful resuscitation of out-of-hospital cardiac arrest: The OHCA score. Eur Heart J. 2006;27(23):2840–5.

9. Maupain C, Bougouin W, Lamhaut L, Deye N, Diehl JL, Geri G, et al. The CAHP (Cardiac Arrest Hospital Prognosis) score: A tool for risk stratification after out-of-hospital cardiac arrest. Eur Heart J. 2016;37(42):3222–8.

10. Martinell L, Nielsen N, Herlitz J, Karlsson T, Horn J, Wise MP, et al. Early predictors of poor outcome after out-of-hospital cardiac arrest. Crit Care. 2017;21(1):1–10.

11. Pareek N, Kordis P, Beckley-Hoelscher N, Pimenta D, Kocjancic ST, Jazbec A, et al. A practical risk score for early prediction of neurological outcome after out-of-hospital cardiac arrest: MIRACLE2. Eur Heart J. 2020;41(47):4508–17.
